# Adrenal androgen mediates the association between obesity and accelerated skeletal maturation in children and adolescents

**DOI:** 10.3389/fendo.2026.1883721

**Published:** 2026-08-18

**Authors:** Youngha Choi, Eunju Lee, Hye Sun Lee, Eun Jae Hwang, Youn Kyoung Kim, Seo Jung Kim, Kyoung Won Cho, Sujin Kim, Kyungchul Song, Junghwan Suh, Eun Byoul Lee, Hyun Wook Chae

**Affiliations:** 1 Department of Pediatrics, Yonsei University College of Medicine, Gangnam Severance Hospital, Seoul, Republic of Korea; 2 Biostatistics Collaboration Unit, Yonsei University College of Medicine, Seoul, Republic of Korea; 3 Department of Pediatrics, Severance Children’s Hospital, Yonsei University College of Medicine, Seoul, Republic of Korea; 4 Department of Pediatrics, Yongin Severance Hospital, Yonsei University College of Medicine, Yongin-si, Republic of Korea

**Keywords:** adolescent, bone age, child, dehydroepiandrosterone sulfate, obesity

## Abstract

**Background:**

Childhood obesity is associated with advanced bone age (BA), but the underlying endocrine mechanisms remain unclear. We investigated the mediating role of dehydroepiandrosterone sulfate (DHEA-S) in the relationship between adiposity and skeletal maturation.

**Methods:**

This retrospective study included 1,252 children aged 4–12 years from a single center. Skeletal maturation was assessed using the BA-to-chronological age (CA) ratio. Advanced maturation was defined as BA/CA > 1.1. Mediation analysis was performed to evaluate whether DHEA-S explains the association between BMI SDS and skeletal maturation.

**Results:**

Body mass index standard deviation score (BMI SDS) and DHEA-S were independently associated with the BA/CA ratio (p < 0.05). In boys younger than 9 years, DHEA-S demonstrated good discriminative performance for advanced maturation (Area under the curve = 0.817). Mediation analysis showed that DHEA-S significantly mediated the association between BMI and BA/CA in boys < 9 years (β = 0.512, p = 0.024) and in girls ≥ 8 years (β = 0.377, p = 0.003). In young boys, the direct effect of BMI became non-significant after inclusion of DHEA-S, consistent with substantial mediation by adrenal androgens.

**Conclusions:**

Adrenal androgen activity, reflected by DHEA-S, represents a significant endocrine pathway linking obesity to accelerated skeletal maturation. The magnitude of this mediation varies by age and sex, highlighting developmentally specific windows of endocrine-metabolic interaction in growing children.

## Introduction

Skeletal maturation is a key determinant of growth potential and pubertal progression in children. Advanced skeletal maturation has been associated with early pubertal onset, rapid linear growth, and premature epiphyseal closure, potentially resulting in reduced adult height (1, 2). Accordingly, bone age (BA) serves as an essential clinical marker in the evaluation of growth and pubertal disorders, and advancement of BA relative to chronological age (CA) is particularly relevant in children with suspected pubertal abnormalities (3, 4). In addition, early pubertal progression is linked to psychosocial challenges, underscoring the broader clinical significance of accelerated skeletal maturation (5, 6).

Childhood obesity is one of the most consistently reported conditions associated with advanced skeletal maturation. Children with overweight or obesity frequently exhibit accelerated BA compared with their normal-weight peers (7, 8). However, the biological mechanisms linking excess adiposity to skeletal maturation remain incompletely defined. Obesity is accompanied by alterations in insulin sensitivity, growth factor dynamics, and androgen metabolism, all of which may influence skeletal development, yet the integrated relationships among adiposity, endocrine status, and pubertal maturation have not been comprehensively evaluated (9–11).

Previous studies examining obesity-related acceleration of skeletal maturation have primarily focused on cross-sectional associations between body mass index (BMI) and BA (7, 8, 11). Although these associations have been reported relatively consistently, clinical and hormonal factors related to skeletal maturation have largely been evaluated in isolation. Some studies have explored clinical or metabolic factors related to skeletal maturation, whereas others have focused on specific hormonal determinants such as insulin-like growth factor-1 (IGF-1) or androgens (8, 12). However, these studies have generally assessed individual factors independently, and relatively few have evaluated the relationships among obesity, clinical or hormonal factors, and skeletal maturation concurrently within a single analytical framework. Moreover, few studies have formally evaluated whether endocrine factors function as mediators linking obesity to skeletal maturation using mediation-based analytical approaches.

Therefore, this study aimed to investigate the association between obesity and skeletal maturation while simultaneously accounting for relevant clinical and hormonal variables. In particular, we sought to examine whether adrenal androgen activity, reflected by serum dehydroepiandrosterone sulfate (DHEA-S) levels, mediates the relationship between adiposity and skeletal maturation. To address this question, we applied mediation analysis to quantify the indirect effect of obesity on skeletal maturation through adrenal androgen pathways.

## Materials and methods

### Ethics statement

This study was reviewed and approved by the Institutional Review Board of Gangnam Severance Hospital (IRB No. 2024-1088-001). The requirement for informed consent was waived due to the retrospective nature of the study and the use of de-identified data.

### Study design and participants

**Figure 1:**
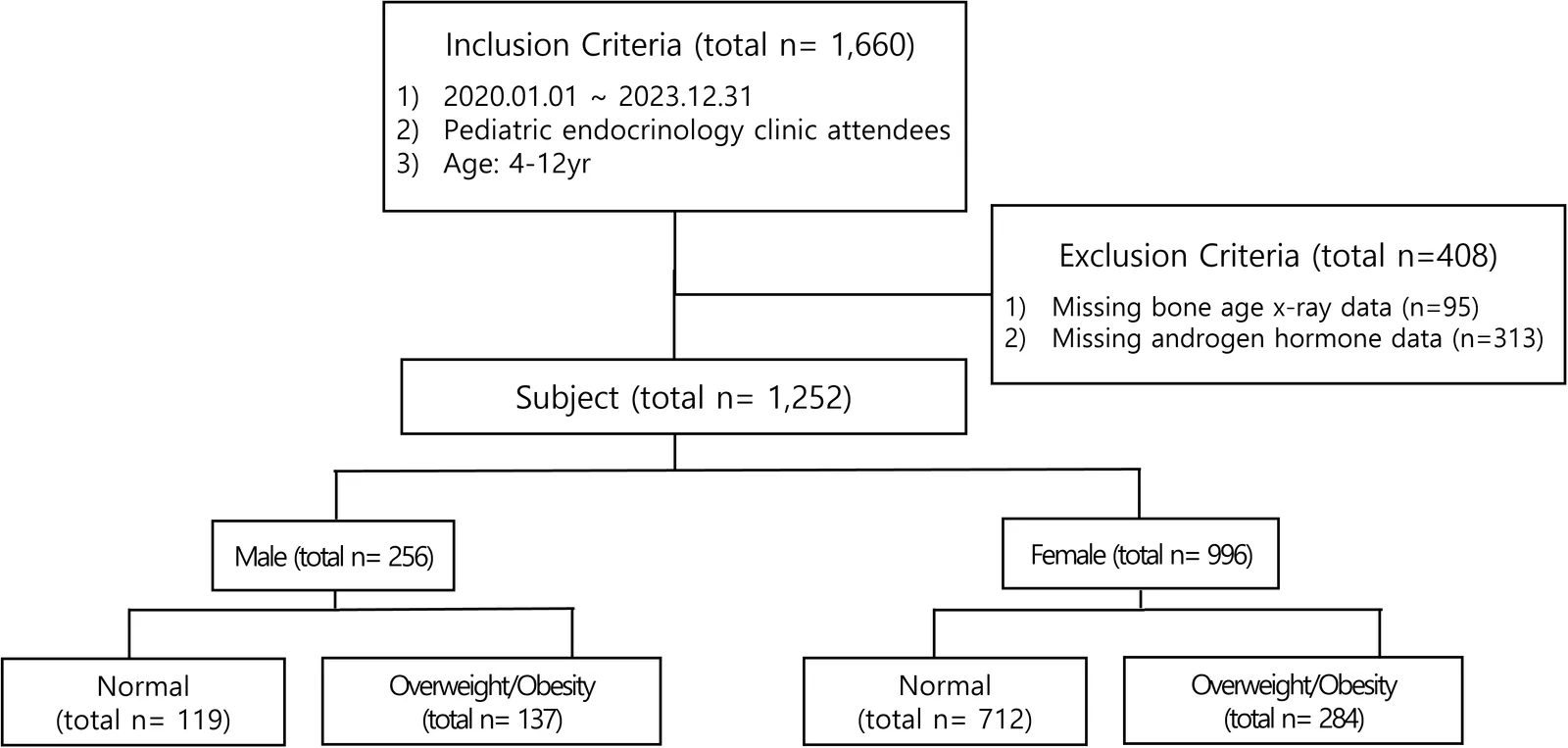
Flowchart of study population selection. Participants were selected from pediatric endocrinology clinic attendees aged 4–12 years. Exclusion criteria included missing key variables and conditions affecting growth or hormonal status.

This retrospective cross-sectional study was conducted at the Department of Pediatric Endocrinology, Yongin Severance Hospital. The study population comprised children aged 4–12 years who attended a pediatric endocrinology outpatient clinic between January 1, 2020, and December 31, 2023. A total of 1,660 children were eligible for the study; however, 408 were excluded due to the absence of BA radiographic data (n = 95) or missing androgen hormone measurements (n = 313). The final analytic sample consisted of 1,252 participants, including 256 boys and 996 girls. Participants were further categorized according to sex and weight status for subgroup analyses. The overall study design and participant selection process are summarized in **Figure 1**.

### Anthropometric measurements

Height was measured to the nearest 0.1 cm using a stadiometer (range: 850–2060 mm; Seriter, Holtain Ltd., Crymych, UK), and body weight was measured to the nearest 0.1 kg using a calibrated electronic scale (DB150; CAS, Yangju, South Korea). Body mass index (BMI) was calculated as weight in kilograms divided by height in meters squared (kg/m²). Height, weight, and BMI were expressed as age- and sex-specific standard deviation scores (SDS) based on the 2017 Korean National Growth Charts (13). Participants were categorized according to BMI percentile into three groups: normal weight (<85th percentile), overweight (85th to <95th percentile), and obese (≥95th percentile). Pubertal development was assessed according to Tanner staging by trained pediatric endocrinologists during clinical examination (14, 15).

### Laboratory measurements

Blood samples were obtained as part of routine clinical evaluation and were not collected under fasting conditions. Samples were promptly centrifuged, and serum and plasma aliquots were stored at −70 °C until analysis. Serum concentrations of luteinizing hormone (LH), DHEA-S, and IGF-1 were measured using an electrochemiluminescence immunoassay on the cobas e801 immunoassay system (Roche Diagnostics GmbH, Mannheim, Germany). Plasma glucose, total cholesterol, high-density lipoprotein cholesterol (HDL), alanine aminotransferase (ALT), and uric acid levels were determined using enzyme-based methods with a Hitachi 7600–120 automated chemistry analyzer (Hitachi, Tokyo, Japan).

### Bone age assessment and skeletal maturation

BA was assessed using left-hand and wrist radiographs according to the Greulich and Pyle-based method by an experienced pediatric endocrinologist (16). CA was calculated at the time of BA assessment, and skeletal maturation was quantified using the BA-to-CA ratio (BA/CA). The BA/CA ratio was evaluated both as a continuous variable and as a categorical variable. Advanced skeletal maturation was defined as a BA/CA ratio greater than 1.1 and was used as a dichotomous outcome in categorical analyses. Age- and sex-stratified analyses used cutoffs of 9 years in boys and 8 years in girls, corresponding to the standard clinical threshold for premature adrenarche, defined by early elevation of adrenal androgens including DHEA-S (17, 18).

### Statistical analysis

All analyses were performed using R version 4.4.1 (R Foundation for Statistical Computing, Vienna, Austria) and SAS version 9.4 (SAS Institute Inc., Cary, NC, USA). Continuous variables are presented as mean ± standard deviation and categorical variables as counts and percentages. Group comparisons were performed using appropriate parametric tests for continuous variables and chi-square tests for categorical variables. Correlation analyses were used to assess associations among skeletal maturation indices, anthropometric parameters, and hormonal variables. Linear and logistic regression analyses were conducted to identify factors associated with skeletal maturation, treating the BA/CA ratio as a continuous and categorical outcome (BA/CA > 1.1), respectively. Covariates were selected based on physiological relevance and data availability; HOMA-IR was excluded from these models due to non-fasting sample collection. Mediation analyses were conducted to evaluate whether serum DHEA-S mediates the association between adiposity and skeletal maturation. In the mediation framework, BMI SDS was specified as the independent variable, the BA/CA ratio as the outcome, and serum DHEA-S as the mediator. Both direct and indirect effects were estimated to quantify the extent to which the association between adiposity and skeletal maturation could be explained by adrenal androgen activity. Statistical significance of the indirect effect was assessed using standard mediation models. To evaluate the robustness of our findings against potential selection bias due to missing data, a sensitivity analysis was performed using Multiple Imputation (MI). Missing values for DHEA-S (24.4%) were handled under the missing at random assumption, generating five imputed datasets, and the mediation analysis was re-estimated using the pooled imputed data. A two-sided P value < 0.05 was considered statistically significant.

## Results

### Baseline characteristics

**Table 1** presents the baseline characteristics of the study population according to BMI category. A total of 1,252 children were included in the analysis, comprising 996 girls and 256 boys. Across BMI categories, height SDS, weight SDS, BMI SDS, Tanner stage, BA–CA difference, BA/CA ratio, serum DHEA-S, ALT, and uric acid levels were higher in the overweight/obesity group. Sex-stratified analyses showed similar patterns in girls. In boys, tanner stage was lower in the overweight/obesity group, and no significant difference in DHEA-S levels was observed between BMI categories. (**Supplementary Table 1**).

**Table 1:** Baseline characteristics stratified by BMI category.

Variables	Normal (n=831)	Overweight/Obesity (n=421)	*p-value
Age	8.33 ± 0.92	8.15 ± 1.06	0.003
Height SDS	0.29 ± 0.96	0.82 ± 0.94	< 0.001
Weight SDS	0.03 ± 0.78	1.75 ± 0.70	< 0.001
BMI SDS	-0.15 ± 0.77	1.96 ± 0.73	< 0.001
Tanner stage	1.94 ± 0.52	2.01 ± 0.64	0.031
dBACA	1.39 ± 0.98	1.74 ± 0.95	< 0.001
BA/CA	1.17 ± 0.13	1.22 ± 0.13	< 0.001
DHEA-S	50.16 ± 37.62	62.75 ± 45.95	< 0.001
LH	0.56 ± 1.18	0.52 ± 0.82	0.473
IGF-1	202.40 ± 68.15	201.89 ± 65.95	0.898
HOMA-IR	3.16 ± 1.27	8.22 ± 10.13	0.482
ALT	12.60 ± 6.69	19.55 ± 17.41	< 0.001
Non-HDL	136.00 ± 55.32	113.96 ± 27.84	0.333
Uric acid	4.00 ± 0.78	4.57 ± 0.94	< 0.001

Values are presented as mean ± standard deviation.

*P value is assessed using Student’s t-test and Chi-square test.

BMI, body mass index; SDS, standard deviation score; dBACA, difference between bone age and chronological age; DHEA-S, dehydroepiandrosterone sulfate; LH, luteinizing hormone; IGF-1, insulin-like growth factor 1; HOMA-IR, homeostasis model assessment of insulin resistance; ALT, alanine aminotransferase; HDL, high-density lipoprotein.

### Associations of BMI with skeletal maturation and adrenal androgen levels

**Table 2** presents the correlation analyses between the BA/CA ratio and anthropometric, hormonal, and metabolic variables. BMI SDS and serum DHEA-S levels showed positive correlations with the BA/CA ratio in the overall population, with similar patterns observed in sex- and age-stratified analyses. In multivariable linear regression analyses adjusting for age, sex, and LH, both BMI SDS and DHEA-S remained independently associated with the BA/CA ratio (**Table 3**). In multivariable logistic regression analyses, higher BMI SDS and higher DHEA-S levels were associated with increased odds of advanced skeletal maturation (**Table 4**). In stratified analyses, BMI SDS was not significantly associated with advanced skeletal maturation in boys younger than 9 years or girls younger than 8 years, whereas DHEA-S remained significantly associated across most subgroups.

**Table 2:** Correlations between BA/CA and clinical variables stratified by sex and age.

Variables	BA/CA									
	Total		Male, Age < 9		Male, Age ≥ 9		Female, Age < 8		Female, Age ≥ 8	
	r (95% CI)	*p-value	r (95% CI)	*p-value	r (95% CI)	*p-value	r (95% CI)	*p-value	r (95% CI)	*p-value
BMI SDS	0.22(0.16-0.27)	< 0.001	0.36(0.19-0.51)	< 0.001	0.35(0.19-0.49)	< 0.001	0.15(0.04-0.25)	0.007	0.26(0.19-0.33)	< 0.001
Tanner stage	0.15(0.09-0.20)	< 0.001	-0.11(-0.29-0.07)	0.228	-0.14(-0.30-0.03)	0.107	0.19(0.08-0.29)	< 0.001	0.37(0.30-0.43)	< 0.001
DHEA-S	0.11(0.05-0.16)	< 0.001	0.37(0.20-0.51)	< 0.001	0.34(0.18-0.48)	< 0.001	0.13(0.02-0.23)	0.018	0.28(0.21-0.35)	< 0.001
LH	0.05(-0.01-0.11)	0.076	-0.09(-0.27-0.09)	0.329	-0.09(-0.25-0.08)	0.298	0.07(-0.04-0.18)	0.194	0.22(0.15-0.30)	< 0.001
IGF-1	0.21(0.16-0.27)	< 0.001	0.12(-0.07-0.29)	0.207	0.02(-0.15-0.19)	0.793	0.23(0.12-0.33)	< 0.001	0.40(0.34-0.47)	< 0.001
Glucose	-0.03(-0.09-0.02)	0.23	-0.19(-0.37-0.01)	0.043	-0.02(-0.19-0.15)	0.779	-0.04(-0.14-0.07)	0.515	0.10(0.02-0.18)	0.01
ALT	0.02(-0.03-0.08)	0.418	0.20(0.01-0.38)	0.036	0.15(-0.03-0.31)	0.094	-0.01(-0.11-0.10)	0.926	0.09(0.01-0.17)	0.022
Non-HDL	-0.08(-0.18-0.01)	0.096	-0.13(-037-0.13)	0.334	-0.02-0.26-0.22)	0.882	-0.03(-0.20-0.15)	0.761	-0.10(-0.25-0.07)	0.241
Uric acid	0.10(0.04-0.16)	< 0.001	0.22(0.03-0.39)	0.022	0.17(0.00-0.33)	0.047	0.10(-0.01-0.21)	0.063	0.19(0.12-0.27)	< 0.001

Correlation coefficients (r) are presented with 95% confidence intervals (CI).

*P values were calculated using Pearson correlation analysis. BA/CA, bone age to chronological age ratio; BMI, body mass index; SDS, standard deviation score; DHEA-S, dehydroepiandrosterone sulfate; LH, luteinizing hormone; IGF-1, insulin-like growth factor 1; ALT, alanine aminotransferase; HDL, high-density lipoprotein; CI, confidence interval.

**Table 3:** Multivariable linear regression analysis for BA/CA ratio stratified by sex and age.

Variables	BA/CA ratio: (BA/CA) *100									
	Total		Male, Age < 9		Male, Age ≥ 9		Female, Age < 8		Female, Age ≥ 8	
	β (95% CI)	*p*-value	β (95% CI)	*p*-value	β (95% CI)	*p*-value	β (95% CI)	*p*-value	β (95% CI)	*p*-value
BMI SDS	2.407(1.868-2.946)	< 0.0001	4.548(2.241-6.856)	0.0002	3.217(1.609-4.824)	0.0001	1.663(0.436-2.890)	0.008	1.837(1.269-2.405)	< 0.0001
Tanner stage	3.686(2.420-4.952)	< 0.0001	-4.428(-11.393-2.536)	0.2104	-2.987(-8.005-2.030)	0.241	5.247(2.186-8.308)	0.0008	5.347(4.221-6.473)	< 0.0001
DHEA-S per 10 units	0.957(0.787-1.126)	< 0.0001	2.033(1.341-2.725)	< 0.0001	0.922(0.537-1.306)	< 0.0001	0.781(0.257-1.306)	0.0036	0.787(0.613-0.960)	< 0.0001
IGF-1	0.047(0.037-0.058)	< 0.0001	0.074(0.003-0.144)	0.0407	0.016(-0.019-0.051)	0.3761	0.074(0.042-0.107)	< 0.0001	0.046(0.038-0.055)	< 0.0001
Glucose	0.000(-0.069-0.070)	0.9923	-0.274(-0.594-0.045)	0.0919	-0.021(-0.244-0.201)	0.8502	-0.047(-0.213-0.118)	0.5723	0.066(-0.002-0.135)	0.056
ALT	0.101(0.043-0.159)	0.0006	0.786(0.135-1.437)	0.0184	0.082(-0.016-0.179)	0.0987	-0.005(-0.166-0.156)	0.9493	0.107(0.036-0.179)	0.0033
Non-HDL	-0.020(-0.060-0.019)	0.3129	-0.050(-0.204-0.104)	0.5207	-0.005(-0.100-0.089)	0.9086	-0.025(-0.121-0.070)	0.6011	-0.008(-0.047-0.030)	0.6633
Uric acid	2.222(1.440-3.003)	< 0.0001	5.317(1.074-9.560)	0.0145	2.401(0.031-4.771)	0.0471	1.710(-0.090-3.511)	0.0625	1.642(0.871-2.413)	< 0.0001

Values are presented as β coefficients with 95% confidence intervals (CI). Models were adjusted for age, sex, and luteinizing hormone (LH) levels. β coefficients represent the change in BA/CA ratio (×100) per unit increase in each independent variable. DHEA-S was analyzed per 10-unit increase. BA/CA, bone age to chronological age ratio; BMI, body mass index; SDS, standard deviation score; DHEA-S, dehydroepiandrosterone sulfate; LH, luteinizing hormone; IGF-1, insulin-like growth factor 1; ALT, alanine aminotransferase; HDL, high-density lipoprotein; CI, confidence interval.

**Table 4:** Multivariable logistic regression analysis for advanced skeletal maturation (BA/CA > 1.1).

Variables	BA/CA (> 1.1)									
	Total		Male, Age < 9		Male, Age ≥ 9		Female, Age < 8		Female, Age ≥ 8	
	OR (95% CI)	*p*-value	OR (95% CI)	*p*-value	OR (95% CI)	*p*-value	OR (95% CI)	*p*-value	OR (95% CI)	*p*-value
BMI SDS	1.452(1.278-1.649)	< 0.0001	1.537(1.122-2.105)	0.0075	1.649(1.237-2.200)	0.0007	1.339(0.978-1.834)	0.0684	1.310(1.085-1.582)	0.005
Tanner stage	1.502(1.123-2.010)	0.0061	0.501(0.208-1.206)	0.1232	0.805(0.371-1.746)	0.5828	1.984(0.929-4.241)	0.077	2.124(1.412-3.193)	0.0003
DHEA-S per 10 units	1.284(1.211-1.362)	< 0.0001	1.422(1.202-1.682)	< 0.0001	1.220(1.109-1.342)	< 0.0001	1.275(1.027-1.584)	0.0279	1.309(1.194-1.436)	< 0.0001
IGF-1	1.010(1.007-1.013)	< 0.0001	1.004(0.995-1.013)	0.351	1.005(0.999-1.011)	0.0807	1.019(1.009-1.030)	0.0004	1.011(1.007-1.016)	< 0.0001
Glucose	1.005(0.990-1.020)	0.5276	0.977(0.941-1.015)	0.2392	0.977(0.945-1.011)	0.1835	1.004(0.964-1.046)	0.8529	1.023(1.000-1.046)	0.0523
ALT	1.021(1.003-1.039)	0.0202	1.094(1.000-1.197)	0.0508	1.015(0.994-1.038)	0.1692	1.003(0.960-1.048)	0.8854	1.024(0.993-1.057)	0.1352
Non-HDL	0.990(0.980-1.000)	0.0463	0.987(0.965-1.009)	0.2274	0.994(0.974-1.014)	0.5539	0.988(0.961-1.017)	0.4119	0.992(0.976-1.008)	0.3435
Uric acid	1.408(1.177-1.684)	0.0002	1.923(1.115-3.315)	0.0186	1.466(1.003-2.142)	0.0481	1.205(0.772-1.881)	0.412	1.306(1.009-1.690)	0.0429

Values are presented as odds ratios (OR) with 95% confidence intervals (CI). Advanced skeletal maturation was defined as BA/CA > 1.1. Models were adjusted for age, sex (in total analyses), and luteinizing hormone (LH) levels. DHEA-S was analyzed per 10-unit increase. BA/CA, bone age to chronological age ratio; BMI, body mass index; SDS, standard deviation score; DHEA-S, dehydroepiandrosterone sulfate; LH, luteinizing hormone; IGF-1, insulin-like growth factor 1; ALT, alanine aminotransferase; HDL, high-density lipoprotein; CI, confidence interval.

### Predictive performance for advanced skeletal maturation

**Figure 2:**
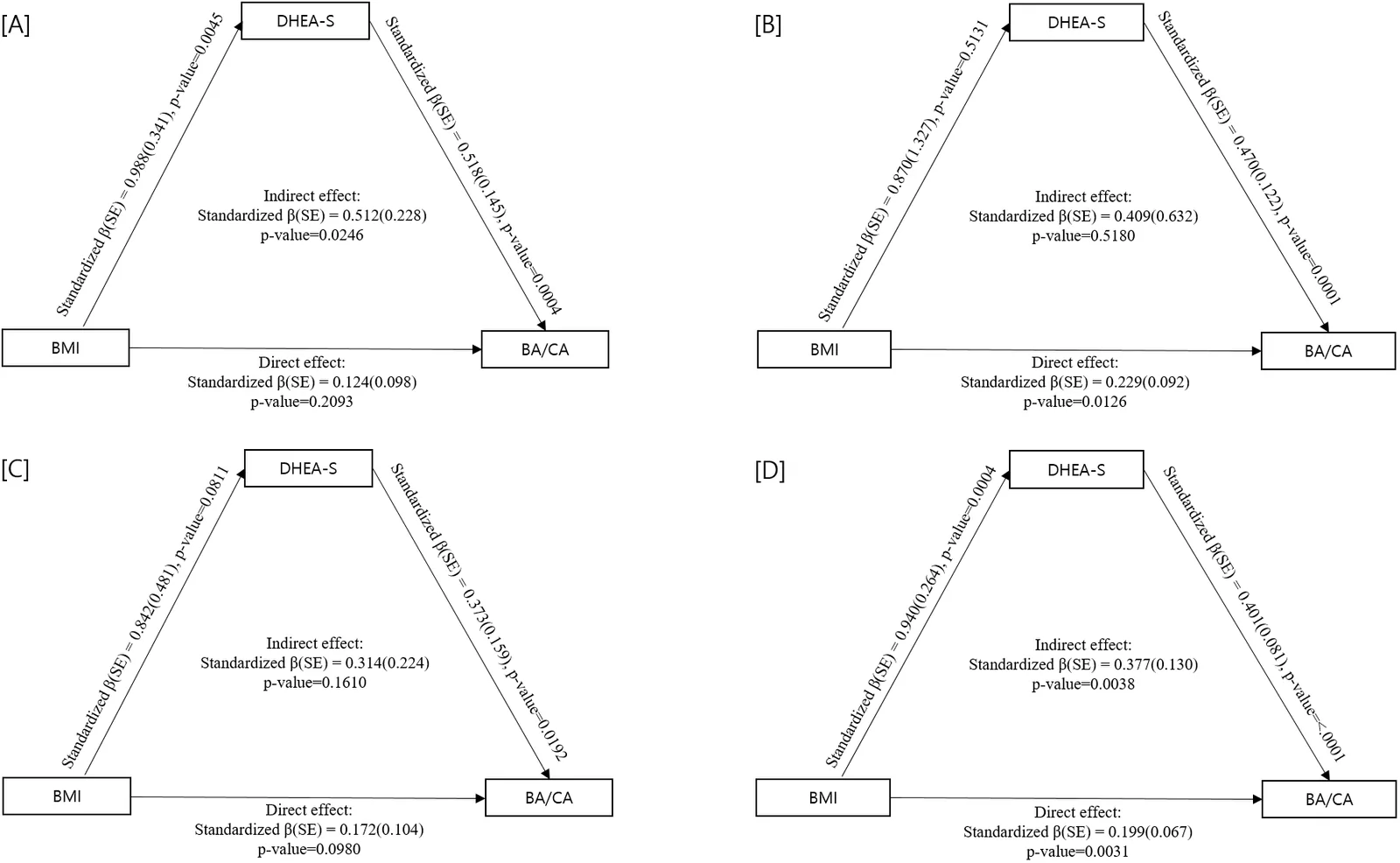
ROC curves of adiposity- and hormone-related markers for predicting advanced skeletal maturation. ROC curve analyses evaluating the discriminative performance of serum DHEA-S for identifying advanced skeletal maturation (defined BA/CA > 1.1). **(A)** Male, < 9 years **(B)** Male, ≥ 9 years **(C)** Female, < 8 years **(D)** Female, ≥ 8 years. ROC, receiver operating characteristic; AUC, area under the curve; BMI SDS, body mass index standard deviation score; IGF-1, insulin-like growth factor 1; HDL, high-density lipoprotein; ALT, alanine aminotransferase; BA, bone age; CA, chronological age ratio; DHEA-S, dehydroepiandrosterone sulfate.

ROC curve analyses were performed to evaluate the discriminative performance of serum DHEA-S for advanced skeletal maturation (BA/CA > 1.1). In the overall population, serum DHEA-S demonstrated moderate discriminative performance, with an area under the curve (AUC) of 0.742. In sex-stratified analyses, the AUC of DHEA-S was 0.772 in boys and 0.716 in girls (**Supplementary Figure 1**). In age-stratified analyses, the AUC was 0.817 in boys younger than 9 years, which was the highest value among the evaluated subgroups, and 0.753 in boys aged 9 years or older. Among girls, the AUC was 0.647 in those younger than 8 years and 0.748 in those aged 8 years or older (**Figure 2**).

### Mediation analysis of obesity, DHEA-S, and skeletal maturation

**Figure 3:**
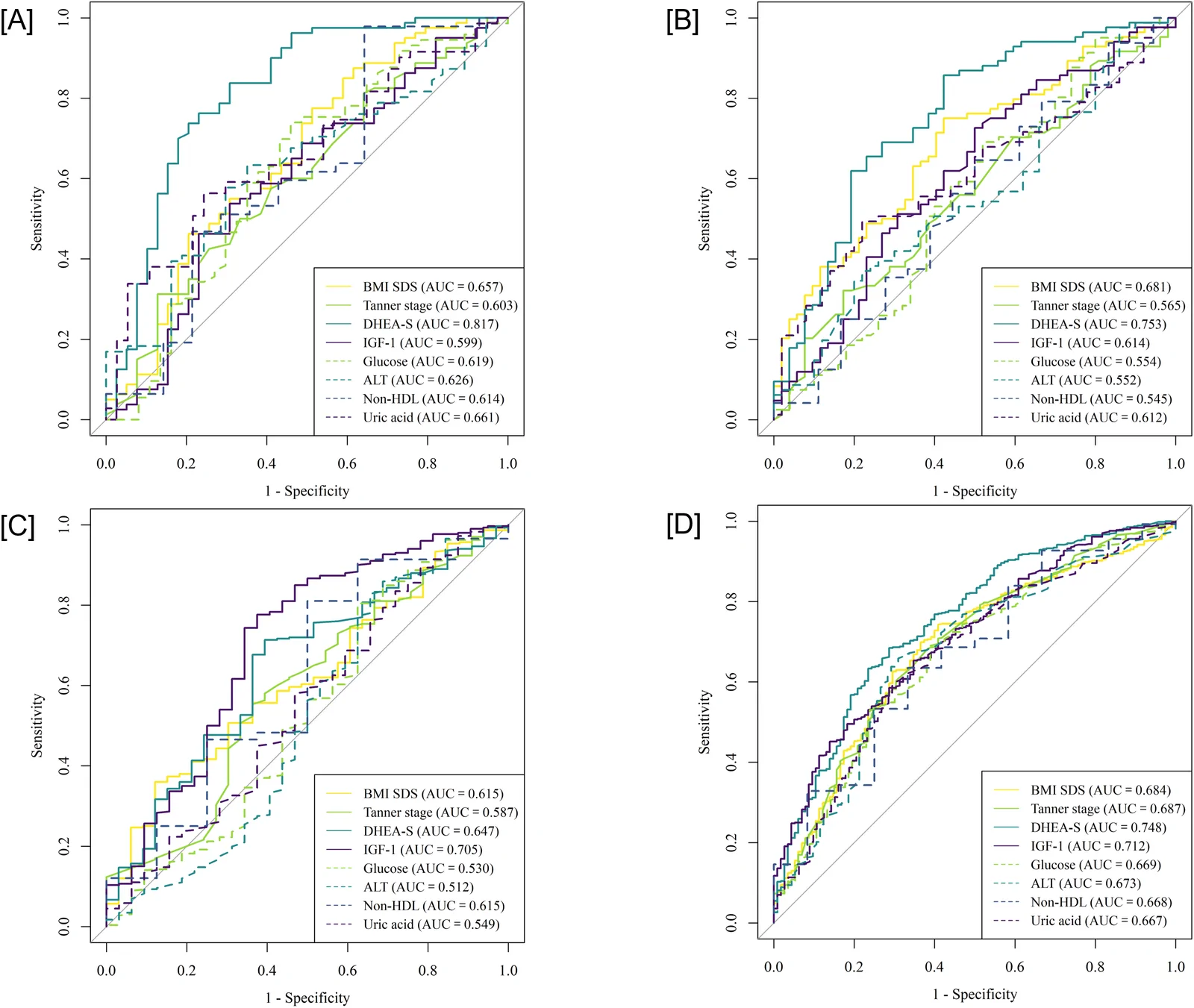
Mediation analysis of DHEA-S in the association between adiposity and skeletal maturation. Mediation analysis was conducted to assess whether DHEA-S mediates the association between adiposity for each pathway. Total, direct, and indirect effects were estimated using adjusted models. Analyses were stratified by sex and age groups. **(A)** Male, < 9 years **(B)** Male, ≥ 9 years **(C)** Female, < 8 years **(D)** Female, ≥ 8 years. BMI, body mass index; BA, bone age; CA, chronological age; DHEA-S, dehydroepiandrosterone sulfate; SE, standard error.

**Figure 3** illustrates the mediation analyses examining the role of serum DHEA-S in the association between BMI and advanced skeletal maturation (BA/CA > 1.1). In the overall population, a significant indirect effect of BMI on skeletal maturation through DHEA-S was observed (standardized β = 0.243, SE = 0.069, p = 0.0004) (**Supplementary Figure 2**). In sex-stratified analyses, the indirect effect was not significant in boys overall but was significant in boys younger than 9 years (standardized β = 0.512, SE = 0.228, p = 0.0246). Among girls, a significant indirect effect was observed in the overall female population (standardized β = 0.292, SE = 0.123, p = 0.0180) and was primarily observed in girls aged 8 years or older (standardized β = 0.377, SE = 0.130, p = 0.0038), whereas no significant mediation was observed in girls younger than 8 years. The proportion of the total effect of BMI on BA/CA mediated by DHEA-S was 80.5% in boys younger than 9 years, 64.1% in boys aged 9 years or older, 64.6% in girls younger than 8 years, and 65.5% in girls aged 8 years or older. Importantly, the sensitivity analysis using multiple imputation (**Supplementary Table 3**) yielded highly consistent results with the primary complete-case analysis, maintaining a robust mediation effect in the overall population (p = 0.0004) and confirming the biological validity of our findings despite missing data.

## Discussion

This study suggests an association between childhood obesity and accelerated skeletal maturation that may be linked to adrenal androgen status, assessed by serum DHEA-S, within a single analytical framework. Both BMI SDS and DHEA-S were independently associated with BA/CA after multivariable adjustment, and higher levels of each were related to increased odds of advanced skeletal maturation. Importantly, DHEA-S showed moderate discriminative performance within this cohort and exerted a significant mediating effect, the magnitude of which varied by sex and age; as this was not a diagnostic accuracy study, these findings should be regarded as hypothesis-generating rather than validated for clinical screening. Childhood obesity has long been associated with accelerated growth and advanced BA (7, 8, 19). Obesity involves alterations in the growth hormone (GH)–IGF axis, hyperinsulinemia, and changes in adipokines. Despite reduced GH secretion, hyperinsulinemia may increase IGF-1 bioavailability and exert direct growth-promoting effects, while experimental studies suggest that insulin-related metabolic signals can stimulate chondrocyte activity and endochondral ossification (20–24). Adipokines such as leptin may further modulate hypothalamic–pituitary function and growth plate activity (25, 26). In our study, although circulating IGF-1 did not differ across BMI categories, it remained independently associated with BA/CA, suggesting that altered hormonal bioactivity or growth plate sensitivity may contribute to BA advancement beyond absolute IGF-1 concentrations. Nevertheless, IGF-1 alone is unlikely to account fully for the observed skeletal acceleration.

Adrenal androgens have been increasingly recognized as potential contributors to skeletal maturation, particularly during the transition from prepuberty to puberty (27–29). The physiological link between excess adiposity and increased adrenal androgen production likely involves complex metabolic–endocrine interactions. Insulin resistance and compensatory hyperinsulinemia, common features of childhood obesity, have been proposed to enhance adrenal androgen synthesis, potentially through insulin receptor substrate 1 and 2 dependent pathways that promote the conversion of Δ^5^ steroids such as DHEA-S to Δ^4^ androgens including androstenedione (30–33). Adipokines such as leptin may also contribute to the regulation of adrenal function and the timing of adrenarche, possibly by stimulating adrenal 17,20-lyase activity and thereby augmenting adrenal androgen production (34, 35).

In line with these proposed mechanisms, our findings provide clinical evidence supporting a mediating role of adrenal androgens in obesity-related skeletal maturation (28, 36, 37). DHEA-S was consistently associated with BA/CA across multivariable models, and mediation analyses demonstrated that DHEA-S significantly mediated the association between BMI and skeletal maturation. Notably, among boys younger than 9 years, the indirect effect remained statistically significant while the direct effect of BMI was attenuated and no longer significant, a pattern consistent with the possibility that adrenal androgen activity may account for a substantial proportion of the obesity–BA association in this subgroup. In contrast, no significant mediation effect was observed in boys aged 9 years or older, whereas in girls, mediation became evident at 8 years or older.

Collectively, our findings suggest that the influence of adrenal androgens on skeletal maturation is both sex- and age-dependent, reflecting distinct developmental windows of endocrine susceptibility. In boys younger than 9 years, the significant mediation by DHEA-S suggests that adiposity-driven adrenarche may represent a predominant pathway linking excess adiposity to accelerated bone maturation, at an age preceding the typical onset of gonadarche when adrenal androgens act relatively unopposed by testicular steroidogenesis (17, 38). In older boys, the increasing influence of gonadal sex steroids, particularly estrogen derived from testicular testosterone, may attenuate the relative contribution of DHEA-S to skeletal maturation (39). Conversely, in girls, the emergence of significant mediation at age 8 years or older may reflect the interplay between adiposity and pubertal steroidogenesis, as adrenarche and early gonadarche tend to overlap temporally in girls, potentially augmented by peripheral aromatization of adrenal precursors (40, 41). Taken together, these findings support the concept of an obesity–adrenal–bone axis as a biologically plausible and developmentally specific endocrine pathway underlying accelerated skeletal maturation.

Despite these strengths, several limitations should be acknowledged. First, the cross-sectional design precludes causal inference; because BMI, DHEA-S, and bone age were measured concurrently, temporal precedence cannot be confirmed, and the mediation findings should be interpreted as statistical associations rather than a proven causal pathway. Longitudinal studies spanning the pubertal transition are warranted. Second, recruitment from a pediatric endocrinology clinic may have introduced referral bias, limiting generalizability to the general pediatric population. Third, approximately one-quarter of participants lacked DHEA-S measurements due to lab omissions at baseline, which could introduce selection bias if missingness was not completely random. To address this, we performed a sensitivity analysis using multiple imputation under the assumption of missing at random (MAR), which yielded mediation results consistent with the complete-case analysis, suggesting our conclusions were unlikely to be materially affected by missingness. However, as with most retrospective clinical datasets, the plausibility of the MAR assumption cannot be directly verified, and a missing not at random mechanism—for instance, selective omission of DHEA-S testing based on unmeasured clinical judgment—cannot be entirely excluded. Fourth, additional adrenal androgens such as androstenedione and 11-oxygenated androgens, as well as metabolic factors such as fasting insulin resistance indices were not assessed, and metabolic parameters were obtained in the non-fasting state, which may have limited precise assessment of insulin resistance and its role in skeletal maturation. Nevertheless, this study has notable strengths, including comprehensive multivariable adjustment, use of BA/CA as a standardized indicator of skeletal advancement, and formal mediation analyses clarifying potential endocrine pathways; age- and sex-stratified analyses further identified developmental windows in which adrenal androgen activity appears particularly relevant.

In conclusion, adrenal androgen activity, particularly DHEA-S, partially mediates the association between childhood obesity and accelerated skeletal maturation in a sex- and age-dependent manner. These findings support the role of the obesity–adrenal–bone axis as a key endocrine pathway underlying skeletal advancement, suggesting that adrenal androgen status may warrant further investigation as a potential marker in the clinical evaluation of children with obesity for advanced skeletal maturation, pending validation in independent cohorts. Further longitudinal studies are needed to clarify causal relationships and long-term outcomes.

## Data Availability

The original contributions presented in the study are included in the article/**Supplementary Material**. Further inquiries can be directed to the corresponding author.

## Supplementary material

Supplementary Figure 1 ROC curves of adiposity- and hormone-related markers for predicting advanced skeletal maturation ROC curves were constructed to evaluate the predictive performance of adiposity- and hormone-related markers for advanced skeletal maturation (defined as BA/CA ≥1.1). Analyses were performed in the total population and stratified by sex. The area under the curve (AUC) was calculated for each model. **(A)** Total population **(B)** Male **(C)** Female. ROC, receiver operating characteristic; AUC, area under the curve; BA, bone age; CA, chronological age; BMI SDS, body mass index standard deviation score; DHEA-S, dehydroepiandrosterone sulfate; IGF-1, insulin-like growth factor 1; HDL, high-density lipoprotein; ALT, alanine aminotransferase.

Supplementary Figure 2 Mediation analysis of DHEA-S in the association between adiposity and skeletal maturation Mediation analysis was conducted to assess whether DHEA-S mediates the association between adiposity (BMI) and skeletal maturation (BA/CA). Standardized regression coefficients (β) with standard errors (SE) and corresponding p-values are presented for each pathway. Total, direct, and indirect effects were estimated using adjusted models. Analyses were performed in the total population and stratified by sex. **(A)** Total population **(B)** Male **(C)** Female. BMI, body mass index; BA, bone age; CA, chronological age; DHEA-S, dehydroepiandrosterone sulfate; SE, standard error.
