# Supplementary material for: Adrenal androgen mediates the association between obesity and accelerated skeletal maturation in children and adolescents
Source: Front Endocrinol (Lausanne). 2026 Aug 18;17:1883721. doi: 10.3389/fendo.2026.1883721 (PMC13531181; doi:10.3389/fendo.2026.1883721)
Supplement: Supplementary file 1 [file Table1.docx]

Supplementary Table 1. Baseline characteristics stratified by BMI category and Sex

|  | Male (n=256) | | | Female (n=996) | | |
| --- | --- | --- | --- | --- | --- | --- |
|  | Normal  (n=119) | Overweight/  Obesity (n=137) | **p*-value | Normal  (n=712) | Overweight/  Obesity (n=284) | **p*-value |
| Age | 8.95±1.18 | 8.67±1.10 | 0.057 | 8.23±0.83 | 7.90±0.94 | < 0.001 |
| Height, cm | 133.32±8.86 | 136.52±8.26 | 0.003 | 129.91±6.95 | 130.34±7.89 | 0.416 |
| Weight, kg | 31.22±5.84 | 43.72±8.85 | < 0.001 | 28.04±4.23 | 36.30±6.77 | < 0.001 |
| BMI SDS | -0.14±0.77 | 2.13±0.80 | < 0.001 | -0.16±0.77 | 1.87±0.68 | < 0.001 |
| Tanner stage | 1.93±0.43 | 1.68±0.51 | < 0.001 | 1.94±0.53 | 2.18±0.63 | < 0.001 |
| dBACA | 0.65±1.38 | 1.43±1.17 | < 0.001 | 1.51±0.84 | 1.89±0.79 | < 0.001 |
| BA/CA | 1.08±0.16 | 1.17±0.15 | < 0.001 | 1.19±0.12 | 1.24±0.11 | < 0.001 |
| DHEA-S | 70.63±56.72 | 82.25±50.09 | 0.083 | 46.74±32.20 | 53.34±40.68 | 0.015 |
| LH | 0.80±1.27 | 0.45±0.40 | 0.005 | 0.52±1.16 | 0.56±0.95 | 0.637 |
| IGF-1 | 191.13±66.53 | 181.13±56.10 | 0.193 | 204.30±68.28 | 211.90±68.06 | 0.113 |
| Glucose | 97.93±13.87 | 95.53±7.34 | 0.101 | 94.23±9.43 | 94.92±9.93 | 0.311 |
| ALT | 14.27±6.58 | 23.31±23.95 | < 0.001 | 12.32±6.68 | 17.83±13.08 | < 0.001 |
| Non-HDL | 132.67±29.01 | 112.60±27.61 | 0.216 | 138.50±74.43 | 114.59±27.98 | 0.566 |
| Uric acid | 4.11±0.84 | 4.76±0.88 | < 0.001 | 3.98±0.77 | 4.49±0.95 | < 0.001 |

Note: Values are presented as mean ± standard deviation.

**P* value is assessed using Student’s t-test and Chi-square test.

Abbreviations: BMI, body mass index; SDS, standard deviation score; dBACA, difference between bone age and chronological age; DHEA-S, dehydroepiandrosterone sulfate; LH, luteinizing hormone; IGF-1, insulin-like growth factor 1; ALT, alanine aminotransferase; HDL, high-density lipoprotein; HDL, high density lipoprotein cholesterol.

Supplementary Table 2. Baseline characteristics of included versus excluded participants

| Variable | Included (n=1,258) | Excluded (n=402) | *p*-value |
| --- | --- | --- | --- |
| Age, yr | 8.28 ± 0.95 | 8.44 ± 1.39 | 0.009 |
| Sex (female), % | 1003 (79.7) | 320 (79.6) | 1.000 |
| Height, cm | 131.13 ± 7.74 | 131.63 ± 11.97 | 0.328 |
| Weight, kg | 31.93 ± 7.77 | 34.36 ± 9.47 | <0.001 |
| BMI SDS | 0.55 ± 1.22 | 0.88 ± 1.31 | <0.001 |
| Tanner stage | 1.96 ± 0.56 | 2.00 ± 0.74 | 0.296 |
| ALT | 14.9 ± 11.9 | **19.2 ± 21.5** | 0.002 |
| Uric acid | **4.20 ± 0.88** | 4.44 ± 1.02 | <0.001 |

Note: Values are presented as mean ± standard deviation or n (%).

**P* value is assessed using Student’s t-test and Chi-square test.

Abbreviations: BMI SDS; body mass index standard deviation score; ALT, alanine aminotransferase.

| Group | Path | CC coef (SE) | *p*-value | MI coef (SE) | *p*-value |
| --- | --- | --- | --- | --- | --- |
| Total | BMI → DHEA-S | 0.956 (0.185) | <0.0001 | 0.937 (0.196) | <0.0001 |
|  | DHEA-S → BA-CA | 0.254 (0.053) | <0.0001 | 0.208 (0.040) | <0.0001 |
|  | BMI → BA-CA (direct) | 0.134 (0.042) | 0.0015 | 0.100 (0.035) | 0.0037 |
|  | Indirect effect | 0.243 (0.069) | 0.0004 | 0.195 (0.055) | 0.0004 |
| Male | BMI → DHEA-S | 0.955 (0.548) | 0.0829 | 0.923 (0.603) | 0.1267 |
|  | DHEA-S → BA-CA | 0.443 (0.091) | <0.0001 | 0.424 (0.076) | <0.0001 |
|  | BMI → BA-CA (direct) | 0.206 (0.068) | 0.0023 | 0.221 (0.059) | 0.0002 |
|  | Indirect effect | 0.422 (0.258) | 0.1012 | 0.391 (0.265) | 0.1400 |
| Female | BMI → DHEA-S | 0.854 (0.317) | 0.0071 | 0.807 (0.331) | 0.0148 |
|  | DHEA-S → BA-CA | 0.342 (0.069) | <0.0001 | 0.218 (0.049) | <0.0001 |
|  | BMI → BA-CA (direct) | 0.229 (0.057) | <0.0001 | 0.138 (0.044) | 0.0015 |
|  | Indirect effect | 0.292 (0.123) | 0.0180 | 0.176 (0.082) | 0.0323 |
| Male, Age<9 | BMI → DHEA-S | 0.988 (0.341) | 0.0045 | 0.987 (0.293) | 0.0010 |
|  | DHEA-S → BA-CA | 0.518 (0.145) | 0.0004 | 0.449 (0.116) | 0.0001 |
|  | BMI → BA-CA (direct) | 0.124 (0.098) | 0.2093 | 0.130 (0.085) | 0.1287 |
|  | Indirect effect | 0.512 (0.228) | 0.0246 | 0.444 (0.174) | 0.0110 |
| Male, Age≥9 | BMI → DHEA-S | 0.870 (1.327) | 0.5131 | 0.725 (1.611) | 0.6532 |
|  | DHEA-S → BA-CA | 0.470 (0.122) | 0.0001 | 0.484 (0.106) | <0.0001 |
|  | BMI → BA-CA (direct) | 0.229 (0.092) | 0.0126 | 0.250 (0.082) | 0.0022 |
|  | Indirect effect | 0.409 (0.632) | 0.5180 | 0.351 (0.783) | 0.6542 |
| Female, Age<8 | BMI → DHEA-S | 0.842 (0.481) | 0.0811 | 0.802 (0.496) | 0.1064 |
|  | DHEA-S → BA-CA | 0.373 (0.159) | 0.0192 | 0.355 (0.101) | 0.0004 |
|  | BMI → BA-CA (direct) | 0.172 (0.104) | 0.0980 | 0.105 (0.081) | 0.1966 |
|  | Indirect effect | 0.314 (0.224) | 0.1610 | 0.285 (0.194) | 0.1415 |
| Female, Age≥8 | BMI → DHEA-S | 0.940 (0.264) | 0.0004 | 0.930 (0.257) | 0.0003 |
|  | DHEA-S → BA-CA | 0.401 (0.081) | <0.0001 | 0.238 (0.057) | 0.0003 |
|  | BMI → BA-CA (direct) | 0.199 (0.067) | 0.0031 | 0.107 (0.051) | 0.0340 |
|  | Indirect effect | 0.377 (0.130) | 0.0038 | 0.222 (0.081) | 0.0062 |

Supplementary Table 3. Comparison of complete-case and multiple imputation–based mediation analyses of the association between BMI, DHEA-S, and BA/CA

Note: Coefficient (SE) and p-value are presented for each path in the mediation model.

Abbreviations: CC, complete-case analysis; MI, multiple imputation; Coef, coefficient; SE, standard error; BMI, body mass index; DHEA-S, dehydroepiandrosterone sulfate; BA/CA, bone age/chronological age ratio.
